# 2-[(*N*-Benzyl-4-methyl­benzene­sul­fon­amido)meth­yl]pyridinium nitrate

**DOI:** 10.1107/S1600536809042330

**Published:** 2009-10-23

**Authors:** Jiang-Sheng Li, Jim Simpson

**Affiliations:** a School of Chemistry and Biological Engineering, Changsha University of Science & Technology, Changsha 410004, People’s Republic of China; b Department of Chemistry, University of Otago, PO Box 56, Dunedin, New Zealand

## Abstract

In the title compound, C_20_H_21_N_2_O_2_S^+^·NO_3_^−^, the dihedral angle between the pyridinium and phenyl rings is 81.77 (19)°, that between the pyridinium and tolyl rings is 1.36 (18)°, and that between the phenyl and tolyl rings is 82.69 (19)°. In the crystal, the components are linked by strong charge-assisted bifurcated N^+^—H⋯(O,O) hydrogen bonds and the packing is consolidated by numerous weak C—H⋯O bonds and π–π stacking inter­actions [for the latter, centroid–centroid separation = 3.868 (2) Å].

## Related literature

For the preparation of the title compound and for a related structure, see: Zhang *et al.* (2007 ▶).
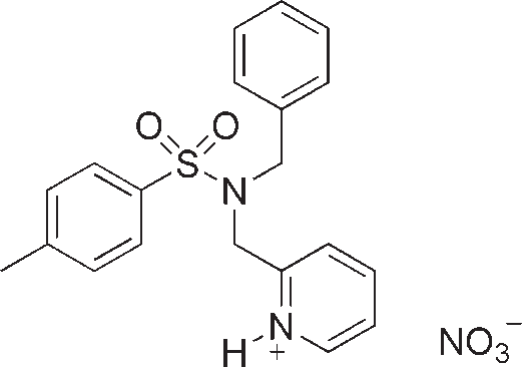


## Experimental

### 

#### Crystal data

C_20_H_21_N_2_O_2_S^+^·NO_3_^−^
*M* *_r_* = 415.46
Triclinic, 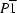

*a* = 7.6852 (15) Å
*b* = 9.811 (2) Å
*c* = 13.240 (3) Å
α = 104.26 (3)°
β = 91.82 (3)°
γ = 95.64 (2)°
*V* = 961.2 (3) Å^3^
*Z* = 2
Mo *K*α radiation
μ = 0.21 mm^−1^
*T* = 113 K
0.20 × 0.18 × 0.12 mm

#### Data collection

Rigaku Saturn CCD diffractometer
Absorption correction: multi-scan (*CrystalClear*; Rigaku/MSC, 2005 ▶) *T*_min_ = 0.960, *T*_max_ = 0.976
7087 measured reflections
3355 independent reflections
2020 reflections with *I* > 2σ(*I*)
*R*_int_ = 0.099

#### Refinement

*R*[*F* ^2^ > 2σ(*F* ^2^)] = 0.077
*wR*(*F* ^2^) = 0.188
*S* = 0.94
3355 reflections
267 parameters
18 restraints
H atoms treated by a mixture of independent and constrained refinement
Δρ_max_ = 0.76 e Å^−3^
Δρ_min_ = −0.73 e Å^−3^

### 

Data collection: *CrystalClear* (Rigaku/MSC, 2005 ▶); cell refinement: *CrystalClear*; data reduction: *CrystalClear*; program(s) used to solve structure: *SHELXS97* (Sheldrick, 2008 ▶); program(s) used to refine structure: *SHELXL97* (Sheldrick, 2008 ▶); molecular graphics: *SHELXTL* (Sheldrick, 2008 ▶) and *Mercury* (Macrae *et al.*, 2006 ▶); software used to prepare material for publication: *SHELXL97*, *enCIFer* (Allen *et al.*, 2004 ▶) and *PLATON* (Spek, 2009 ▶).

## Supplementary Material

Crystal structure: contains datablocks global, I. DOI: 10.1107/S1600536809042330/hb5137sup1.cif

Structure factors: contains datablocks I. DOI: 10.1107/S1600536809042330/hb5137Isup2.hkl

Additional supplementary materials:  crystallographic information; 3D view; checkCIF report

## Figures and Tables

**Table 1:** Hydrogen-bond geometry (Å, °)

*D*—H⋯*A*	*D*—H	H⋯*A*	*D*⋯*A*	*D*—H⋯*A*
N1—H1*A*⋯O3	0.91 (4)	1.82 (4)	2.670 (5)	156 (3)
N1—H1*A*⋯O4	0.91 (4)	2.39 (4)	3.161 (5)	144 (3)
C6—H6*B*⋯O3	0.99	2.66	3.362 (5)	128
C15—H15⋯O4	0.95	2.68	3.493 (5)	144
C1—H1⋯O2^i^	0.95	2.71	3.315 (5)	122
C3—H3⋯O1^ii^	0.95	2.60	3.301 (5)	131
C20—H20*A*⋯O3^iii^	0.98	2.43	3.256 (5)	142
C7—H7*A*⋯O4^iv^	0.99	2.70	3.440 (5)	132
C11—H11⋯O4^v^	0.95	2.54	3.439 (5)	158
C11—H11⋯O5^v^	0.95	2.55	3.411 (5)	151
C20—H20*C*⋯O3^vi^	0.98	2.62	3.594 (5)	174
C10—H10⋯O5^vii^	0.95	2.66	3.309 (6)	126

Symmetry codes: (i) 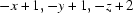; (ii) 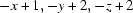; (iii) 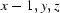; (iv) 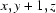; (v) 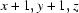; (vi) 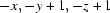; (vii) 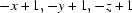.

**Fig. 1.**
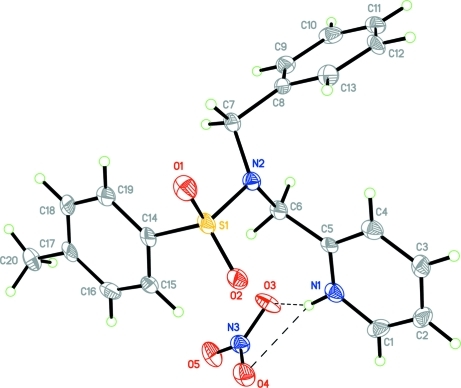
The structure of (I) showing displacement ellipsoids drawn at the 30% probability level and H atoms shown as spheres of arbitrary radius.

**Fig. 2.**
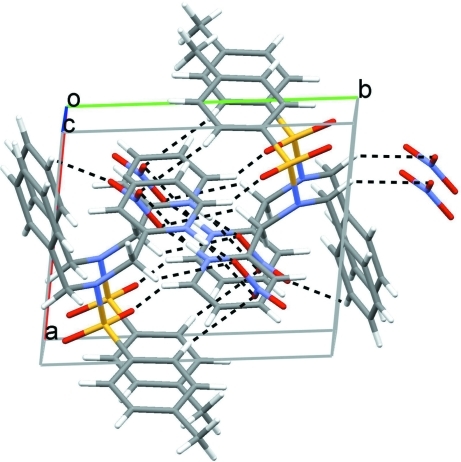
Crystal packing of (I). Hydrogen bonds are indicated as dashed lines.

**Fig. 3.**
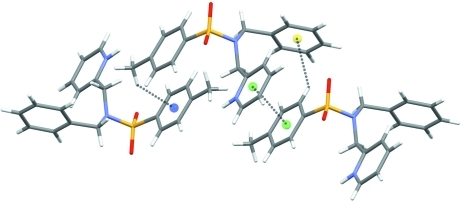
Crystal packing of (I) viaπ-π and C—H···π interactions, indicated as dashed lines.

## supplementary crystallographic information

### Comment

The molecular structure of (I) (Fig. 1) shows that the nitrate is connected with its corresponding pyridinium *via* two strong charge-assisted N^+^—H···O hydrogen bonds. In the cation, the dihedral angle between the pyridinium and phenyl rings is 81.774 (9)°, that between the pyridinium and tolyl rings 1.355 (5)°, and that between the phenyl and tolyl rings 82.693 (7)°.

In the crystal structure, a series of intermolecular C—H···O interactions link the molecules (Table 1), Fig. 2, which are packed by π-π stacking interactions between the pyridinium ring and the tolyl ring at (1 + *x*, *y*, *z*) [centroid-to -centroid separation 3.868 Å], together with two weak C—H···π interactions [H19···*Cg*2(*x* - 1, *y*, *z*) 2.90 Å, H20B···*Cg*3(-*x*, *y*, *z*) 2.69 Å; *Cg*2 and *Cg*3 are the centroids of the phenyl and tolyl rings,respectively], Fig.3.

### Experimental

The tosylamino-containing pyridine derivative was prepared by a similar method to that of Zhang *et al.* (2007). Colourless needles of (I) were obtained by natural evaporation from its aqueous nitric acid solution.

### Refinement

The N-bound H atom was located in a difference map and refined with the distance restraint N—H = 0.91 (4) Å. The other H atoms were positioned geometrically and constrained to ride on their parent atoms [C—H distances are 0.95 and 0.99Å for aromatic and CH_2_ H atoms with *U*_iso_(H) = 1.2 *U*_eq_(C), 0.98 Å, *U*_iso_ = 1.5*U*_eq_ (C) for CH_3_ atoms.

### Crystal data

**Table:**

C_20_H_21_N_2_O_2_S^+^·NO_3_^−^	*Z* = 2
*M**_r_* = 415.46	*F*(000) = 436
Triclinic, *P*1	*D*_x_ = 1.435 Mg m^−^^3^
Hall symbol: -P 1	Mo *K*α radiation, λ = 0.71073 Å
*a* = 7.6852 (15) Å	Cell parameters from 2706 reflections
*b* = 9.811 (2) Å	θ = 2.2–27.9°
*c* = 13.240 (3) Å	µ = 0.21 mm^−^^1^
α = 104.26 (3)°	*T* = 113 K
β = 91.82 (3)°	Cut needle, colourless
γ = 95.64 (2)°	0.20 × 0.18 × 0.12 mm
*V* = 961.2 (3) Å^3^	

### Data collection

**Table:**

Rigaku Saturn CCD diffractometer	3355 independent reflections
Radiation source: fine-focus sealed tube	2020 reflections with *I* > 2σ(*I*)
graphite	*R*_int_ = 0.099
Detector resolution: 7.31 pixels mm^-1^	θ_max_ = 25.0°, θ_min_ = 1.6°
ω and φ scans	*h* = −9→9
Absorption correction: multi-scan (*CrystalClear*; Rigaku/MSC, 2005)	*k* = −11→10
*T*_min_ = 0.960, *T*_max_ = 0.976	*l* = −13→15
7087 measured reflections	

### Refinement

**Table:**

Refinement on *F*^2^	Primary atom site location: structure-invariant direct methods
Least-squares matrix: full	Secondary atom site location: difference Fourier map
*R*[*F*^2^ > 2σ(*F*^2^)] = 0.077	Hydrogen site location: inferred from neighbouring sites
*wR*(*F*^2^) = 0.188	H atoms treated by a mixture of independent and constrained refinement
*S* = 0.94	*w* = 1/[σ^2^(*F*_o_^2^) + (0.0858*P*)^2^] where *P* = (*F*_o_^2^ + 2*F*_c_^2^)/3
3355 reflections	(Δ/σ)_max_ = 0.002
267 parameters	Δρ_max_ = 0.76 e Å^−^^3^
18 restraints	Δρ_min_ = −0.73 e Å^−^^3^

### Special details

**Table:**

Geometry. All e.s.d.'s (except the e.s.d. in the dihedral angle between two l.s. planes) are estimated using the full covariance matrix. The cell e.s.d.'s are taken into account individually in the estimation of e.s.d.'s in distances, angles and torsion angles; correlations between e.s.d.'s in cell parameters are only used when they are defined by crystal symmetry. An approximate (isotropic) treatment of cell e.s.d.'s is used for estimating e.s.d.'s involving l.s. planes.
Refinement. Refinement of *F*^2^ against ALL reflections. The weighted *R*-factor *wR* and goodness of fit *S* are based on *F*^2^, conventional *R*-factors *R* are based on *F*, with *F* set to zero for negative *F*^2^. The threshold expression of *F*^2^ > σ(*F*^2^) is used only for calculating *R*-factors(gt) *etc*. and is not relevant to the choice of reflections for refinement. *R*-factors based on *F*^2^ are statistically about twice as large as those based on *F*, and *R*- factors based on ALL data will be even larger.

### Fractional atomic coordinates and isotropic or equivalent isotropic displacement parameters (Å^2^)

**Table:**

	*x*	*y*	*z*	*U*_iso_*/*U*_eq_	
S1	0.18143 (12)	0.79196 (10)	0.86138 (8)	0.0205 (3)	
O1	0.1109 (3)	0.9220 (3)	0.9041 (2)	0.0274 (7)	
O2	0.2282 (3)	0.7040 (3)	0.9273 (2)	0.0246 (7)	
N1	0.5863 (4)	0.5427 (4)	0.8435 (3)	0.0227 (8)	
N2	0.3629 (4)	0.8340 (3)	0.8070 (2)	0.0182 (8)	
C1	0.6827 (5)	0.4979 (4)	0.9147 (3)	0.0263 (10)	
H1	0.6861	0.3995	0.9078	0.032*	
C2	0.7735 (5)	0.5931 (4)	0.9951 (3)	0.0256 (10)	
H2	0.8407	0.5620	1.0449	0.031*	
C3	0.7679 (5)	0.7362 (4)	1.0043 (3)	0.0240 (10)	
H3	0.8321	0.8043	1.0599	0.029*	
C4	0.6674 (5)	0.7786 (4)	0.9313 (3)	0.0234 (10)	
H4	0.6620	0.8764	0.9368	0.028*	
C5	0.5758 (5)	0.6794 (4)	0.8510 (3)	0.0178 (9)	
C6	0.4652 (5)	0.7138 (4)	0.7666 (3)	0.0204 (9)	
H6A	0.5421	0.7370	0.7132	0.024*	
H6B	0.3836	0.6294	0.7322	0.024*	
C7	0.3525 (5)	0.9325 (4)	0.7392 (3)	0.0225 (10)	
H7A	0.2762	1.0060	0.7695	0.027*	
H7B	0.3007	0.8802	0.6693	0.027*	
C8	0.5338 (5)	1.0014 (4)	0.7292 (3)	0.0190 (9)	
C9	0.6059 (5)	0.9836 (4)	0.6327 (3)	0.0246 (10)	
H9	0.5395	0.9303	0.5715	0.030*	
C10	0.7760 (5)	1.0438 (4)	0.6253 (4)	0.0285 (11)	
H10	0.8249	1.0324	0.5591	0.034*	
C11	0.8726 (5)	1.1195 (4)	0.7142 (4)	0.0277 (11)	
H11	0.9891	1.1589	0.7094	0.033*	
C12	0.8006 (5)	1.1386 (4)	0.8104 (4)	0.0254 (10)	
H12	0.8675	1.1913	0.8716	0.030*	
C13	0.6312 (5)	1.0808 (4)	0.8175 (3)	0.0232 (10)	
H13	0.5813	1.0957	0.8836	0.028*	
C14	0.0365 (5)	0.6861 (4)	0.7588 (3)	0.0182 (9)	
C15	0.0291 (5)	0.5396 (4)	0.7362 (3)	0.0219 (10)	
H15	0.0953	0.4958	0.7786	0.026*	
C16	−0.0733 (5)	0.4593 (4)	0.6532 (3)	0.0250 (10)	
H16	−0.0801	0.3591	0.6390	0.030*	
C17	−0.1692 (5)	0.5217 (4)	0.5878 (3)	0.0224 (10)	
C18	−0.1592 (5)	0.6684 (4)	0.6119 (3)	0.0222 (10)	
H18	−0.2239	0.7125	0.5689	0.027*	
C19	−0.0576 (5)	0.7516 (4)	0.6967 (3)	0.0224 (10)	
H19	−0.0522	0.8518	0.7122	0.027*	
C20	−0.2769 (5)	0.4302 (4)	0.4942 (3)	0.0288 (10)	
H20A	−0.3691	0.3705	0.5174	0.043*	
H20B	−0.2014	0.3703	0.4488	0.043*	
H20C	−0.3302	0.4904	0.4557	0.043*	
H1A	0.525 (5)	0.470 (4)	0.796 (3)	0.014 (10)*	
O3	0.4546 (4)	0.3632 (3)	0.6655 (2)	0.0349 (8)	
O4	0.2927 (4)	0.2835 (3)	0.7733 (2)	0.0319 (8)	
N3	0.3338 (4)	0.2729 (3)	0.6806 (3)	0.0258 (8)	
O5	0.2626 (4)	0.1803 (3)	0.6083 (2)	0.0346 (8)	

### Atomic displacement parameters (Å^2^)

**Table:**

	*U*^11^	*U*^22^	*U*^33^	*U*^12^	*U*^13^	*U*^23^
S1	0.0123 (5)	0.0245 (6)	0.0255 (6)	0.0017 (4)	0.0065 (4)	0.0070 (4)
O1	0.0220 (16)	0.0240 (15)	0.0345 (19)	0.0057 (12)	0.0082 (14)	0.0020 (13)
O2	0.0183 (15)	0.0334 (17)	0.0247 (18)	−0.0012 (12)	0.0055 (13)	0.0132 (13)
N1	0.0161 (19)	0.022 (2)	0.030 (2)	0.0022 (15)	0.0065 (16)	0.0068 (17)
N2	0.0156 (11)	0.0195 (11)	0.0202 (11)	0.0022 (8)	0.0038 (8)	0.0057 (8)
C1	0.026 (2)	0.025 (2)	0.034 (3)	0.0112 (19)	0.008 (2)	0.015 (2)
C2	0.018 (2)	0.036 (3)	0.029 (3)	0.0085 (19)	0.005 (2)	0.018 (2)
C3	0.013 (2)	0.034 (3)	0.026 (3)	0.0035 (18)	0.0050 (18)	0.011 (2)
C4	0.019 (2)	0.022 (2)	0.031 (3)	0.0034 (17)	0.009 (2)	0.0079 (19)
C5	0.0084 (18)	0.019 (2)	0.030 (2)	0.0035 (15)	0.0100 (17)	0.0117 (17)
C6	0.013 (2)	0.022 (2)	0.028 (3)	0.0050 (16)	0.0076 (18)	0.0076 (18)
C7	0.012 (2)	0.024 (2)	0.034 (3)	0.0021 (17)	0.0049 (19)	0.0121 (19)
C8	0.012 (2)	0.020 (2)	0.030 (3)	0.0036 (16)	0.0044 (18)	0.0120 (18)
C9	0.021 (2)	0.023 (2)	0.033 (3)	0.0046 (18)	0.004 (2)	0.0118 (19)
C10	0.025 (2)	0.028 (2)	0.040 (3)	0.0091 (19)	0.016 (2)	0.020 (2)
C11	0.013 (2)	0.024 (2)	0.052 (3)	0.0042 (18)	0.010 (2)	0.019 (2)
C12	0.010 (2)	0.020 (2)	0.046 (3)	−0.0011 (16)	0.003 (2)	0.010 (2)
C13	0.018 (2)	0.025 (2)	0.029 (3)	0.0073 (18)	0.009 (2)	0.0077 (19)
C14	0.0093 (18)	0.024 (2)	0.023 (2)	0.0040 (15)	0.0103 (16)	0.0066 (17)
C15	0.015 (2)	0.026 (2)	0.028 (3)	0.0012 (17)	0.0058 (19)	0.0132 (19)
C16	0.024 (2)	0.020 (2)	0.033 (3)	−0.0006 (18)	0.009 (2)	0.0090 (19)
C17	0.010 (2)	0.033 (2)	0.026 (3)	0.0011 (17)	0.0111 (18)	0.0100 (19)
C18	0.010 (2)	0.033 (2)	0.028 (3)	0.0080 (17)	0.0102 (18)	0.0120 (19)
C19	0.015 (2)	0.023 (2)	0.032 (3)	0.0068 (17)	0.0122 (19)	0.0081 (19)
C20	0.019 (2)	0.034 (2)	0.031 (3)	−0.0003 (18)	0.007 (2)	0.005 (2)
O3	0.0331 (18)	0.0320 (17)	0.039 (2)	−0.0090 (14)	0.0143 (15)	0.0111 (14)
O4	0.0257 (17)	0.0449 (19)	0.0253 (19)	0.0004 (13)	0.0091 (14)	0.0092 (14)
N3	0.021 (2)	0.027 (2)	0.033 (2)	0.0045 (16)	0.0084 (17)	0.0113 (18)
O5	0.0264 (17)	0.0375 (18)	0.032 (2)	−0.0073 (14)	0.0016 (15)	−0.0019 (15)

### Geometric parameters (Å, °)

**Table:**

S1—O1	1.429 (3)	C9—H9	0.9500
S1—O2	1.431 (3)	C10—C11	1.376 (6)
S1—N2	1.652 (3)	C10—H10	0.9500
S1—C14	1.773 (4)	C11—C12	1.383 (6)
N1—C5	1.331 (5)	C11—H11	0.9500
N1—C1	1.362 (5)	C12—C13	1.384 (5)
N1—H1A	0.91 (4)	C12—H12	0.9500
N2—C7	1.478 (5)	C13—H13	0.9500
N2—C6	1.481 (4)	C14—C15	1.388 (5)
C1—C2	1.351 (6)	C14—C19	1.389 (5)
C1—H1	0.9500	C15—C16	1.358 (6)
C2—C3	1.384 (5)	C15—H15	0.9500
C2—H2	0.9500	C16—C17	1.406 (5)
C3—C4	1.385 (5)	C16—H16	0.9500
C3—H3	0.9500	C17—C18	1.389 (5)
C4—C5	1.371 (6)	C17—C20	1.505 (6)
C4—H4	0.9500	C18—C19	1.380 (6)
C5—C6	1.507 (5)	C18—H18	0.9500
C6—H6A	0.9900	C19—H19	0.9500
C6—H6B	0.9900	C20—H20A	0.9800
C7—C8	1.512 (5)	C20—H20B	0.9800
C7—H7A	0.9900	C20—H20C	0.9800
C7—H7B	0.9900	O3—N3	1.275 (4)
C8—C13	1.384 (5)	O4—N3	1.258 (4)
C8—C9	1.388 (6)	N3—O5	1.217 (4)
C9—C10	1.398 (5)		
			
O1—S1—O2	120.51 (18)	C8—C9—C10	120.1 (4)
O1—S1—N2	106.19 (16)	C8—C9—H9	119.9
O2—S1—N2	106.00 (16)	C10—C9—H9	119.9
O1—S1—C14	109.31 (17)	C11—C10—C9	119.8 (4)
O2—S1—C14	107.56 (17)	C11—C10—H10	120.1
N2—S1—C14	106.42 (17)	C9—C10—H10	120.1
C5—N1—C1	121.9 (4)	C10—C11—C12	120.2 (4)
C5—N1—H1A	125 (2)	C10—C11—H11	119.9
C1—N1—H1A	113 (2)	C12—C11—H11	119.9
C7—N2—C6	114.8 (3)	C11—C12—C13	120.0 (4)
C7—N2—S1	116.9 (2)	C11—C12—H12	120.0
C6—N2—S1	114.4 (2)	C13—C12—H12	120.0
C2—C1—N1	120.2 (4)	C8—C13—C12	120.5 (4)
C2—C1—H1	119.9	C8—C13—H13	119.7
N1—C1—H1	119.9	C12—C13—H13	119.7
C1—C2—C3	119.5 (4)	C15—C14—C19	120.8 (4)
C1—C2—H2	120.3	C15—C14—S1	120.0 (3)
C3—C2—H2	120.3	C19—C14—S1	119.1 (3)
C2—C3—C4	119.1 (4)	C16—C15—C14	119.7 (4)
C2—C3—H3	120.5	C16—C15—H15	120.1
C4—C3—H3	120.5	C14—C15—H15	120.1
C5—C4—C3	120.0 (4)	C15—C16—C17	121.2 (4)
C5—C4—H4	120.0	C15—C16—H16	119.4
C3—C4—H4	120.0	C17—C16—H16	119.4
N1—C5—C4	119.3 (4)	C18—C17—C16	118.0 (4)
N1—C5—C6	116.2 (4)	C18—C17—C20	121.9 (3)
C4—C5—C6	124.4 (3)	C16—C17—C20	120.1 (3)
N2—C6—C5	112.7 (3)	C19—C18—C17	121.5 (4)
N2—C6—H6A	109.1	C19—C18—H18	119.2
C5—C6—H6A	109.1	C17—C18—H18	119.2
N2—C6—H6B	109.1	C18—C19—C14	118.8 (4)
C5—C6—H6B	109.1	C18—C19—H19	120.6
H6A—C6—H6B	107.8	C14—C19—H19	120.6
N2—C7—C8	109.7 (3)	C17—C20—H20A	109.5
N2—C7—H7A	109.7	C17—C20—H20B	109.5
C8—C7—H7A	109.7	H20A—C20—H20B	109.5
N2—C7—H7B	109.7	C17—C20—H20C	109.5
C8—C7—H7B	109.7	H20A—C20—H20C	109.5
H7A—C7—H7B	108.2	H20B—C20—H20C	109.5
C13—C8—C9	119.3 (4)	O5—N3—O4	121.9 (4)
C13—C8—C7	119.8 (4)	O5—N3—O3	121.1 (4)
C9—C8—C7	120.8 (4)	O4—N3—O3	117.0 (4)
			
O1—S1—N2—C7	−47.2 (3)	C7—C8—C9—C10	−177.7 (3)
O2—S1—N2—C7	−176.5 (3)	C8—C9—C10—C11	0.7 (5)
C14—S1—N2—C7	69.2 (3)	C9—C10—C11—C12	−1.3 (5)
O1—S1—N2—C6	174.5 (3)	C10—C11—C12—C13	0.3 (6)
O2—S1—N2—C6	45.2 (3)	C9—C8—C13—C12	−1.8 (5)
C14—S1—N2—C6	−69.1 (3)	C7—C8—C13—C12	176.7 (3)
C5—N1—C1—C2	−1.0 (6)	C11—C12—C13—C8	1.3 (5)
N1—C1—C2—C3	−0.1 (6)	O1—S1—C14—C15	−151.3 (3)
C1—C2—C3—C4	0.7 (6)	O2—S1—C14—C15	−18.9 (4)
C2—C3—C4—C5	−0.2 (6)	N2—S1—C14—C15	94.4 (3)
C1—N1—C5—C4	1.5 (6)	O1—S1—C14—C19	34.0 (4)
C1—N1—C5—C6	179.7 (3)	O2—S1—C14—C19	166.4 (3)
C3—C4—C5—N1	−0.8 (6)	N2—S1—C14—C19	−80.3 (3)
C3—C4—C5—C6	−178.9 (4)	C19—C14—C15—C16	−0.9 (6)
C7—N2—C6—C5	140.3 (3)	S1—C14—C15—C16	−175.5 (3)
S1—N2—C6—C5	−80.4 (3)	C14—C15—C16—C17	1.5 (6)
N1—C5—C6—N2	141.3 (3)	C15—C16—C17—C18	−1.3 (6)
C4—C5—C6—N2	−40.5 (5)	C15—C16—C17—C20	178.0 (4)
C6—N2—C7—C8	−62.9 (4)	C16—C17—C18—C19	0.5 (6)
S1—N2—C7—C8	158.9 (3)	C20—C17—C18—C19	−178.7 (4)
N2—C7—C8—C13	−59.5 (4)	C17—C18—C19—C14	0.1 (6)
N2—C7—C8—C9	119.0 (4)	C15—C14—C19—C18	0.1 (6)
C13—C8—C9—C10	0.8 (5)	S1—C14—C19—C18	174.8 (3)

### Hydrogen-bond geometry (Å, °)

**Table:**

*D*—H···*A*	*D*—H	H···*A*	*D*···*A*	*D*—H···*A*
N1—H1A···O3	0.91 (4)	1.82 (4)	2.670 (5)	156 (3)
N1—H1A···O4	0.91 (4)	2.39 (4)	3.161 (5)	144 (3)
C6—H6B···O3	0.99	2.66	3.362 (5)	128
C15—H15···O4	0.95	2.68	3.493 (5)	144
C1—H1···O2^i^	0.95	2.71	3.315 (5)	122
C3—H3···O1^ii^	0.95	2.60	3.301 (5)	131
C20—H20A···O3^iii^	0.98	2.43	3.256 (5)	142
C7—H7A···O4^iv^	0.99	2.70	3.440 (5)	132
C11—H11···O4^v^	0.95	2.54	3.439 (5)	158
C11—H11···O5^v^	0.95	2.55	3.411 (5)	151
C20—H20C···O3^vi^	0.98	2.62	3.594 (5)	174
C10—H10···O5^vii^	0.95	2.66	3.309 (6)	126

Symmetry codes: (i) −*x*+1, −*y*+1, −*z*+2; (ii) −*x*+1, −*y*+2, −*z*+2; (iii) *x*−1, *y*, *z*; (iv) *x*, *y*+1, *z*; (v) *x*+1, *y*+1, *z*; (vi) −*x*, −*y*+1, −*z*+1; (vii) −*x*+1, −*y*+1, −*z*+1.
